# Advancing AI Data Ethics in Nursing: Future Directions for Nursing Practice, Research, and Education

**DOI:** 10.2196/62678

**Published:** 2024-10-25

**Authors:** Patricia A Ball Dunlap, Martin Michalowski

**Affiliations:** 1 School of Nursing, University of Minnesota, 5-140 Weaver-Densford Hall, 308 Harvard Street SE, Minneapolis, MN, 55455, United States, 16126245959; 2 Center for Digital Health, Mayo Clinic, Rochester, MN, United States

**Keywords:** artificial intelligence, AI data ethics, data-centric AI, nurses, nursing informatics, machine learning, data literacy, health care AI, responsible AI

## Abstract

The ethics of artificial intelligence (AI) are increasingly recognized due to concerns such as algorithmic bias, opacity, trust issues, data security, and fairness. Specifically, machine learning algorithms, central to AI technologies, are essential in striving for ethically sound systems that mimic human intelligence. These technologies rely heavily on data, which often remain obscured within complex systems and must be prioritized for ethical collection, processing, and usage. The significance of data ethics in achieving responsible AI was first highlighted in the broader context of health care and subsequently in nursing. This viewpoint explores the principles of data ethics, drawing on relevant frameworks and strategies identified through a formal literature review. These principles apply to real-world and synthetic data in AI and machine-learning contexts. Additionally, the data-centric AI paradigm is briefly examined, emphasizing its focus on data quality and the ethical development of AI solutions that integrate human-centered domain expertise. The ethical considerations specific to nursing are addressed, including 4 recommendations for future directions in nursing practice, research, and education and 2 hypothetical nurse-focused ethical case studies. The primary objectives are to position nurses to actively participate in AI and data ethics, thereby contributing to creating high-quality and relevant data for machine learning applications.

## Introduction

Artificial intelligence (AI) has become increasingly popular in the United States and globally. Major US media outlets frequently report on AI, covering topics from job displacement concerns to its diverse and innovative applications across various industries. In the health care sector, where there is a vast amount of electronic administrative and clinical data, the adoption and application of AI technology are expected to grow significantly, with projections suggesting a market increase to approximately US$208 million by 2030 and a compounded annual growth rate of 38.5% worldwide [1]. Moreover, AI ethics has gained more recognition due to the negative outcomes and ethical issues related to algorithmic bias, lack of transparency, trust issues, data security, and fairness. A preliminary examination of a global AI ethics case registry indicates that AI incidents proliferate across multiple industry sectors [2].

Machine learning algorithms, particularly those featured in headlines or academic papers about ethical violations, are often central to these discussions. For instance, large language models and other machine learning algorithms have been reported to generate incorrect medical results [3-5], exhibit bias [5,6], and fail to identify chronically ill Black American patients who require high-risk care management [5,7]. These reports effectively draw attention to the malfunctioning of AI technologies. However, deeper analysis reveals that the underlying root cause of these failures often lies in the data used by these algorithms. Data integrity is fundamental to AI technology, as it powers these influential systems [8-10].

Poor quality or unrepresentative data in developing AI technologies can lead to significant issues, such as generative AI algorithms producing incorrect responses (known as “hallucinations”) and the degradation of machine learning model performance when encountering new data. These problems can disrupt operations and damage the public reputation of organizations, particularly in high-stakes environments like health care, thereby endangering patient outcomes and safety. Thus, data must be prioritized and scrutinized in discussions about AI, emphasizing the importance of ethical data collection, processing, and usage. Data ethics are critical for developing well-designed AI solutions and achieving responsible AI in health care, especially in technologies tailored for nursing.

Progressing from the existing literature, this viewpoint paper explores the principles of data ethics and their critical role in achieving responsible AI in health care and nursing. It also presents existing ethical data frameworks and strategies, emphasizing how data operations and usage contribute to generating high-quality data sets. These data sets are essential for training AI technologies to meet their intended performance and value objectives. The objectives of our viewpoints are to (1) introduce the principles of data ethics and relevant frameworks within the health care context; (2) examine how data-centric AI methodologies uphold the principles of data ethics by fostering the creation of high-quality data sets for machine learning; and (3) discuss the importance of AI data ethics, data-centric AI, and data quality for nursing practice, education, and research, including offering recommendations for future directions. We advocate further exploration and discussion of AI data ethics among nurses and nurse informatics researchers. With an effective organizational data governance structure and committed data-centric culture, we hypothesize that data ethics will significantly enhance AI development quality, thereby facilitating ethical and responsible AI solutions in practice, education, and research.

## Responsible AI

Before discussing AI data ethics in depth, it is pertinent to introduce the concept of responsible AI, as its principles greatly influence those of AI data ethics. Responsible AI aims to ensure that AI systems are designed, developed, and deployed in ethical, fair, transparent, accountable, and beneficial ways to all intended users [11]. It is a multidimensional approach aiming to establish standards and values that prevent security issues, biases, and discrimination [12]. Five principles underpin the creation of responsible AI solutions and they are (1) accountability; (2) sustainability; (3) bias, fairness, and privacy; (4) transparency and explainability; and (5) robustness, security, and safety [13,14]. These principles also inform the principles of AI data ethics.

## AI Data Ethics

### About AI Data Ethics and Its Role in AI

Data ethics is a critical aspect of responsible AI focusing on the ethical aspects of data operations, including data collection, processing, and use. It encompasses a broad range of ethical issues related to data handling, whether the data are structured or unstructured, across various modalities [15-17]. Existing research indicates that poor data ethics can have significant consequences. For instance, 57% of consumers reported they would stop doing business with companies that breached their trust through irresponsible data practices [15]. Moreover, using suboptimal data to train machine learning algorithms can result in harmful outcomes for patients, such as misdiagnosis [18], misidentification [18,19], or data privacy breaches, even if the data were deidentified before use in machine learning algorithms [8].

In the context of this viewpoint report, it is crucial to analyze data independently from the machine learning algorithms that process it. This separation underscores the need for strategies, governance, a data-literate organizational culture, and ethical principles that ensure the creation and use of high-quality data for AI technologies. As Radziwill [20] noted, human error is often the root cause of bad data. Data may also reflect historical human biases embedded during their production, consumption, curation, and management. Therefore, data ethics is essential for addressing issues and unintended consequences stemming from poorly managed data.

Furthermore, there is a growing recognition of the importance of distinguishing data ethics from AI ethics, allowing for ethical decisions, specifically in data handling and the AI algorithms that use this data [16,17,21]. This approach emphasizes a data-centric perspective that focuses on the moral dimensions of data [16] and the ethical principles governing real-world and synthetic data [22].

### Ethical Concerns Around Real-World and Synthetic Data in AI

Generative and predictive machine learning algorithms require extensive data sets to achieve performance objectives. Data scarcity has emerged as a significant concern within the AI and research communities, primarily due to the depletion of real-world training and validation data sets necessary for AI development. Several factors contribute to this issue, such as website owners increasingly protecting their data by enforcing data consent requirements [23]. It is projected that data scarcity could become a critical issue between 2026 and 2032 [24]. Additionally, the use of real-world data for AI development encounters problems such as missing data, which demands either imputation or deletion.

The generation of synthetic data is being accelerated to address issues related to data scarcity, privacy, and consent [22]. Synthetic data are expected to surpass real-world data by 2030 [22,25]. In the health care sector, synthetic data are used for simulation and prediction research, health IT development, education, and training [22,26]. Despite its benefits, generating synthetic data for AI development presents ethical dilemmas and risks, especially in high-stakes areas such as health care. For instance, although synthetic data can help represent diverse populations and reduce algorithmic biases, overreliance on such data can lead to challenges and unforeseen long-term effects of converting unrepresentative data into representative data [22]. Thus, ethical concerns arise regarding the non-maleficence and fidelity of synthetic data—whether it can address real-world disparities or prevent the dissemination of misinformation [22].

### Principles of AI Data Ethics

Rhem [8] identified eight principles of AI data ethics, which are summarized as (1) transparency: is there clarity regarding the use, purpose, storage, and protection of the collected data? (2) Fairness: does the data collection and usage avoid exacerbating existing inequalities or biases? (3) Privacy: does the data collection process respect individuals’ privacy and autonomy, potentially through informed consent? (4) Responsibility: are data collectors and users accountable for ethical data collection and usage, including any harm resulting from these processes? Are mitigation steps in place? (5) Security: are data stored and transmitted securely to prevent unauthorized access, use, or disclosure? (6) Inclusivity: does the data collection and usage process ensure the inclusion of diverse perspectives and experiences, especially those that are underrepresented? (7) Transparency in decision-making: are decisions based on explainable and interpretable data? (8) Continual assessment: does the organization continuously monitor and assess its data practices to ensure they align with ethical principles [8]?

Shanley et al [22] were inspired by the five principles of responsible AI to initiate discussions on data ethics and synthetic data in AI. They proposed five principles to govern synthetic data (1) responsibility; (2) non-maleficence; (3) privacy; (4) transparency; and (5) justice, fairness, and equity [22]. These principles closely align with the globally recognized principles of AI—responsibility, non-maleficence, privacy, transparency, and justice and fairness [27]. Moreover, the ethical principles suggested by Shanley et al [22] and their associated questions correspond with Rhem’s [8] AI data ethical principles. This alignment is detailed in Table 1, which includes an adaptation of Rhem’s [8] principle of fairness.

**Table 1.** Summary of data ethical principles for AI^a^.

Principles of AI data ethics	Rhem [8] questions	Shanley et al [22] questions	New addition
Transparency	Transparency: is there clarity regarding the use, purpose, storage, and protection of the collected data?	Transparency: how well does the synthetic data capture the phenomena it supposedly represents? How does the synthetic data deviate from the “real” data? What were the considerations when mitigating biases and how were they mitigated?	N/A^b^
Just, fair, and equitable data operations (DataOps)	Fairness: does the data collection and usage avoid exacerbating existing inequalities or biases?	Justice, fairness, and equity: are the underrepresented group’s diversity and emerging novelties adequately considered? How are the developers held accountable for watching new characteristics, traits, or phenomena emerging within the synthetic data set? What is the process of alerting developers of overreliance on synthetic data for groups or populations where data collection is more challenging or costly?	N/A
Privacy	Privacy: does the data collection process respect individuals’ privacy and autonomy, potentially through informed consent?	Privacy: what data privacy policies need to be used for synthetic data set generation and use, including who is responsible for the policies? How do we obtain meaningful consent from the individuals and communities impacted by using their data for synthetic data set generation? What notions of data ownership should pertain to synthetic data set creation?	N/A
Responsibility	Responsibility: are data collectors and users accountable for ethical data collection and usage, including any harm resulting from these processes? Are mitigation steps in place?	Responsibility: who decides when and for what purpose synthetic data set generation is justified? When are real-world data necessary, and when is it appropriate to partially apply synthetic data sets?Suppose synthetic data entails accounting for additional considerations during the decision-making process. Does its use imply new or different responsibilities for those involved in the AI supply value chain? What does this mean for the roles, responsibilities, and decision-making processes of those involved in generating and using the synthetic data?	N/A
Security	Security: are data stored and transmitted securely to prevent unauthorized access, use, or disclosure?	N/A	N/A
Inclusivity	Inclusivity: does the data collection and usage process ensure the inclusion of diverse perspectives and experiences, especially those that are underrepresented?	Non-maleficence: what is the gap between the real world in which the AI is intended to operate and the synthetic world in which it was trained? What means and measures can we use to describe the gap adequately? And what vocabulary can we use to make sense of the uncollected data in the real world regarding its status vis-à-vis knowledge or truth claims? What is the potential for intentional or unintentional misuse of synthetic data?	N/A
Transparency in decision-making	Transparency in decision-making: are decisions based on explainable and interpretable data?	Transparency: see the transparency principle that is provided at the beginning of the table.	N/A
Continual assessment	Continual assessment: does the organization continuously monitor and assess its data practices to ensure they align with ethical principles?	N/A	N/A
Safety	N/A	N/A	Do data operation activities and processes consider, identify, and mitigate risks associated with preventing danger, risk, or injury to individuals (patients)?

a AI: artificial intelligence.

b N/A: not applicable.

Patient safety is paramount in health care and nursing, encompassing the protection of patients from events such as danger, risk, or injury. Security concerns freedom from danger or threats, whereas privacy analyzes the unauthorized access or use of patient data. We suggest incorporating a ninth principle, safety, into Rhem’s [8] 8 principles of AI data ethics, as outlined in Table 1. A pertinent question for this new principle is “Do data operation activities and processes consider, identify, and mitigate risks associated with preventing danger, risk, or injury to individuals (patients)?” There is a dearth of consensus in the scientific and industrial literature from AI and health informatics research communities regarding definitions and practices that would guide AI data ethics. These definitions and shared principles are crucial for guiding the implementation and assessment of data ethics in clinical practice. As noted by Panai [21], data ethics represents an underdeveloped area within organizations and lacks a clear definition. Similarly, AI data ethics is a latent or underexplored area in health care and nursing informatics scientific research.

### Ethical Data Frameworks

Numerous proposals exist for ethical data frameworks that support these principles. Floridi and Taddeo [16] advocate for a macroethical data ethics framework, which aims to avoid narrow, ad-hoc approaches and enables organizations to develop solutions that optimize the societal benefits of data science. Furthermore, Marcovitch and Rancourt [17] endorse standardized tools that facilitate the integration of data ethics accountability mechanisms, such as disclosure and transparency processes, at the organizational level. This is particularly important given the variations in legal frameworks across countries. Their proposed tools include the integration of data ethics into organizational culture, the establishment of data processes or management systems, a data governance structure, organizational transparency in ethical decision-making regarding the data supply chain, and a consistent method for demonstrating and verifying ethical data practices [17].

Note that the frameworks proposed by Floridi and Taddeo [16] and Marcovitch and Rancourt [17] are not specifically tailored to the context of health care or nursing. The absence of a verifiable theoretical or conceptual AI data ethics framework that supports research, alongside a practical evaluation framework that translates effectively into practice, represents a significant gap in health and nursing informatics research. The ideas presented by Floridi and Taddeo [16] and Marcovitch and Rancourt [17] could serve as a foundation for developing such frameworks within the nursing field.

### Ethical Data Frameworks Challenges

Without a shared organizational data vision, strategy, and policies, implementing aspects of the proposed data ethical frameworks may be challenging, including risking data integrity. There needs to be clarity and knowledge about the responsibilities and liabilities of the people in charge of the data processes. The accountability and culture change should begin with the organization’s executive team. Then, the executive team members intelligibly communicate the data cultural expectations, and policy changes to their departments, units, and teams. There is clarity about the responsibilities and liabilities of people who produce and consume the organizational data assets.

Nurses are producers and consumers of organizational data assets. For instance, they produce electronic health record (EHR) data and use them for nursing quality improvement initiatives. Positive deviance in effective data practices could be the impetus for remarkable cultural changes when poor data management practices are in place due to the absence of an organizational-level data vision, strategy, and policies. Suppose a nurse leader is passionate about the societal benefits of data science and AI, including taking the initiative to understand the importance of data quality. This individual becomes a change agent for their team. Their team’s culture becomes data-centric, with patient safety and outcomes at the forefront. The team develops policies and standardized procedures that facilitate improved EHR data entry processes, which are less burdensome for the nurse but help them be accountable to the principles of AI data ethics. The team expresses knowledge about how data quality impacts patient care and the technologies they use in clinical settings. They observe technology as a mechanism to provide quality nursing care. Their attitudes and behaviors result in improved data quality for the nursing unit. This team is now a data vanguard. Other departments and nursing teams notice that this nursing unit outputs high-quality data, leading to improved insights for the unit. This “positive deviant” team’s data practices led to informative unit-level reports, fewer data-related errors, and enhanced patient outcomes. Other nursing teams are curious and want to model this team’s effective data procedures and practices. This influence could motivate the organization’s nursing departments to follow suit.

## Data-Centric AI

### Model-Centric and Data-Centric AI Paradigms

AI technologies that leverage machine learning require substantial data for effective functioning. Machine learning is a subset of AI that enables computers to learn and adapt autonomously through algorithms and statistical models, with minimal or no human intervention. These systems demand extensive data volumes, with generative AI requiring even larger data sets and greater computing power to discern underlying patterns in the data. Historically, the development of prevalent AI technologies has adopted a model-centric strategy, prioritizing the machine learning algorithm or model as the primary focus for enhancing performance [28]. In this approach, data-related activities such as curation, collection, and labeling are often deprioritized and occur only once, leading to potential ethical issues such as algorithmic bias and mispredictions.

The model-centric approach does not adequately address the principles of data ethics, as it overlooks the complexity, nuances, challenges, and accuracy of data, which are vital for improving the behavior of machine learning models [29]. Recognizing these limitations, the AI industry is shifting toward a data-centric strategy, which places data—the “fuel” of AI—at the core of the development process [28-30]. This strategy emphasizes the importance of data quality to achieve high-performance machine learning models [9,29]. Unlike the model-centric approach, data activities in the data-centric strategy are iterative, while model optimization remains static [9,29]. This approach also promotes the involvement of domain experts to secure relevant, high-quality data sets for machine learning [30].

### Data-Centric AI Role in Achieving Data Ethics and Responsible AI

The data-centric AI methodology involves developing, iterating, tracking, and maintaining the quality and integrity of AI systems’ data. It focuses on creating adequate training data, designing appropriate inference data, and ensuring data sustainability (establishing data lineage) [29]. Given the importance of high-quality data, the data-centric approach aligns with the principles of data ethics and responsible AI. For instance, meticulous data curation and collection support ethical principles of fairness, responsibility, and transparency. The involvement of domain experts such as clinicians, clinical informaticists, and regulatory and privacy specialists promotes inclusivity, transparent decision-making, and privacy protection. These diverse perspectives help safeguard patient data privacy and prevent harm from substandard data. Considering data sets as a distinct and valuable product separate from the machine learning model also encourages health care organizations to establish continuous assessment protocols for their data collection and usage practices.

## Significance of Data-Centric AI and AI Data Ethics in Nursing

In the broader health care context, we integrated the principles of data ethics and related data-centric AI strategies into nursing practices, emphasizing their role in creating high-quality data sets for machine learning. We now explore the significance of these concepts in nursing. First, AI has become increasingly prevalent in both nursing research and practice. A recent scoping review highlighted various AI activities and applications within nursing [31]. Nurse researchers and informaticists need to understand the distinctions between model-centric and data-centric AI [32] and their impacts on developing safe and effective AI technologies in health care settings, which influence care processes and workflows. This paper aims to establish a consensus on AI data ethics in nursing and motivate nurse informatics researchers to investigate further and discuss this crucial topic.

Second, numerous machine learning algorithms in health care use data from EHRs. Nurses, who are primary users of EHRs [33,34], generate various electronic clinical documents detailing different aspects of patient care and progress (eg, admission assessments, nursing care plans, nursing education, and medication administration). We hypothesize that most frontline nurses are unaware of how their EHR data influences the performance and use of AI technologies. There is an existing awareness of the negative consequences of poorly designed EHR user interfaces on data quality in research and practice [35-38]. To effectively use EHR data in developing AI-driven clinical decision support systems, data collection and processing by the EHR user community must be optimized [35]. Integrating AI data ethics into both practice and academic nursing curricula could enhance awareness about how EHR data are used by downstream systems and the role nurses play in generating high-quality EHR data for AI technologies. This approach introduces new research opportunities, such as examining the relationships between nurses’ data literacy and AI-related data quality.

Third, nurses who are more aware of data-centric AI and the principles of data ethics could increase their confidence in collaborating with data scientists, engineers, and other AI specialists on data-centric projects to produce meaningful, high-quality data and data sets. These efforts lead to development of high-performance machine learning models that align with nurses’ workflows. This applies to both real-world and synthetic data used in machine learning. Nurses provide valuable insights into patient safety and privacy and understand data collection at the point of care. Their contributions are crucial in helping health care organizations adhere to the principles of AI data ethics, develop responsible AI technologies that enhance their workflows, and support safe patient care.

## Nursing Practice and Research Implications

Machine learning algorithms, such as generative AI algorithms trained on poor-quality and unrepresentative data sets, can create significant political consequences in health care, such as exacerbating bias and health disparities. AI data ethics and data-centric AI represent emerging concepts in nursing. The nursing literature on these topics is limited, including discussions on the implications of using synthetic data to develop AI technologies tailored for nursing.

Finally, fictional nurse-oriented ethical case studies are presented in Textboxes 1 and 2. They illustrate potential ethical data breaches in real-world scenarios. These case studies can help nurses become informed about why AI data ethics should matter to them and examine their data practices, ensuring they perform their best in not becoming unknowingly enablers of data issues but high-data quality contributors and problem-solvers.

Textbox 1. **Fictional nurse-specific case study #1: real-world data.**

**Electronic health record (EHR) burnout leading to poor data entry with adverse data cascade effects**

Ava is a new graduate nurse in her fourth month of orienting on a busy adult medical-surgical unit. Six hours into her third consecutive 12-hour night shift, she must complete electronic clinical documentation for 4 patients in the EHR. Ava is sleep-deprived and highly stressed as she adjusts to becoming a competent med-surg nurse who can function without the supervision of a nurse preceptor. Additionally, her patient load was intense during this shift. She received a new admission from the telemetry floor about a few hours ago, a 70-year-old male patient named Carl. Carl arrived at Ava’s unit moderately agitated and had a newly placed trach. Ava is ready to end her shift and get the much-needed respite. She must complete assessment documentation for Carl before the shift changes. The copy-forward feature was leveraged to accelerate Carl’s assessment documentation, essentially copying some data elements previously documented by a telemetry nurse in the EHR. A data entry error embedded in the previous admission note is unknown to Ava. There is a mistake concerning Carl’s medical history. In the telemetry admission note, the patient’s smoking status was mistakenly documented as “Non-smoker,” although a relative communicated that Carl currently smokes. This incorrect value was pulled into Ava’s shift assessment note via the copy-forward function. Moreover, due to exhaustion, Ava did not realize her assessment notes became bloated with irrelevant and duplicate data because of the copy-forward option. The shift assessment documentation copy-forward action occurred repeatedly by subsequent nurses caring for Carl during his hospitalization.

A few years later, a data scientist is requested to build a new machine learning model that will predict patients having a history of smoking and at risk for moderate to severe mental instability during their hospitalizations because of nicotine withdrawal and other socioeconomic factors. Furthermore, the final artificial intelligence (AI) solution will generate nursing care plans and patient education recommendations. This project was inspired by nurses’ desires to provide equitable care, taking precautionary steps to ensure these patients are comfortable and safe during their hospital stays. The erroneous data tied to Carl’s previous hospital encounter were included in the training data set. An expert nurse was not engaged in the data collection and the data set validation processes.

**AI data ethical principle breach and brief commentary**:

- Responsibility: which data owners or consumers are accountable for the insufficient EHR data used to develop the machine learning model? What are the mitigation steps for mispredictions or generative AI hallucinations that may result in patient harm?
- Inclusivity: how are we assured that the data sets represent the use case and intended patient population? This case study presents missing perspectives from nurse stakeholders, patient advocates, and critical decision makers.
- Safety: a patient encounter with characteristics like Carl’s data is predicted to be low risk, and hence, no recommendations were made by the AI solution. An expert nurse may catch the misprediction and take corrective steps. However, an inexperienced nurse may trust the prediction. This is a missed opportunity, and the proper treatment may be delayed or denied, potentially leading to an adverse patient outcome and reputational harm for the organization.

Textbox 2. **Fictional nurse-specific case study #2: synthetic data.**

**Generating synthetic data for health equity machine learning**

A data scientist is developing a nurse-specific machine learning model to predict whether patients are at risk of nonadherence to cardiac care at-home instructions, increasing their readmission risks. The discharge nurse provides and discusses these instructions with the patient before they are discharged from the hospital to home. The anonymized training and validation data sets have 500 and 300 observations, respectively, derived from the hospital’s electronic health record (EHR) data. The data sets are not representative of a diverse patient population. This concern is significant to the data scientist because the data sets incorporate social drivers of health data elements (SDOH). After all, the project is funded by a federal grant with an initiative to improve health equity among disadvantaged patients in the United States. So, the data set must represent a diverse patient population. No formal organizational artificial intelligence (AI) data ethical policies or data ethics oversight committee exists to guide synthetic data creation. The data scientist does their best to add between 30 and 50 fictional observations, using the available training and validation data sets to guide the synthetic data modeling.

**AI data ethical principle breach and brief commentary**:

- Just, fair, and equitable data operations: the data scientist worked alone to create an assumable diverse data set. This decision should involve multiple key stakeholders and a vetting process to ensure the organization does not experience reputational harm and to protect patients from adverse outcomes. The potentially biased data set used to train the machine learning model may result in unforeseen algorithmic bias.
- Responsibility: if an adverse patient outcome occurs because a nurse trusted the predictions made by this machine learning model, who is held responsible? What does the root cause analysis process look like?
- Inclusivity: the “diverse” data set the data scientist developed may be insufficient and unrepresentative of the intended target population. What policies are guiding the decision around what is considered inclusive data? There are missing perspectives from key stakeholders.
- Transparency in decision-making: can the data scientist adequately explain the decisions behind developing the data sets to nontechnical users? After making a prediction, does the AI solution provide interpretable results that could guide nurses about how it arrived at its conclusion?
- Safety: has the data scientist documented the data activities, including the identified risks to nurses and their patients and the risks’ mitigation plans?

## Recommendations for Nursing

We present 4 recommendations to enable nurses to engage with and contribute to developing responsible AI technologies that align with their workflows and adhere to the principles of AI data ethics. Implementation of the recommendations could transform nursing care, practice, and education around data. They aim to prepare nurses for their future in practicing and learning in health care AI. Transformations include (1) AI technologies effectively reduce nurses’ burdensome documentation, (2) AI and data-literate nurses experiencing a reduction in technological fears like job displacement—rather than fearing the technology, learn and use it to their advantage, (3) nurses advocating for their profession and patients by getting involved in the design of AI technologies, and (4) nurses’ improved data knowledge and management practices leading to insights that positively impact patient outcomes and the service they provide to their patients.

### Recommendation 1: Data Ethics Engagement Necessitates Data Literacy in AI

As AI becomes increasingly integral to the daily responsibilities of nurses across various clinical and administrative settings, nurse leadership must promote a data-centric culture within the nursing profession. Leaders should serve as role models, emphasizing the importance of data quality in nursing practice. One approach to achieving this is by enhancing nurses’ understanding of AI and data. Nurses need to comprehend how downstream systems use the data they produce in EHR. It is crucial to reflect, correct, and evolve from the existing cultural norms and power dynamics that hinder effective EHR data collection, establishing a new culture that recognizes the importance of maintaining data quality at the point of care.

Additionally, nurse educators and researchers should strive to improve their AI and data literacy skills and develop new curricula to bridge the knowledge gap in AI and data among nursing students, particularly those enrolled in nurse informatics programs or those pursuing research in informatics. Data literacy is the ability to explore, read, write, understand, and communicate meaningfully within a specific context [39,40].

Improved data literacy facilitates discussions regarding data usage and ethics in AI. Establishing a common language that clearly defines data literacy and ethics in nursing is necessary, including the principles of AI data ethics to guide the development of nurse-specific AI technologies. Furthermore, a shared language and a solid foundation in data knowledge equip nurses to act as data stewards, engage in ethical discussions, innovate in data management, and collaborate with AI specialists to develop responsible, nurse-specific AI technologies. These initiatives align with the principles of inclusivity and responsibility in AI data ethics.

### Recommendation 2: Data-Oriented Culture Motivates Ethical Accountability Mechanisms

Creating and nurturing a data-oriented culture among nurses can enhance their involvement in ethical accountability mechanisms for AI data, as outlined by Marcovitch and Rancourt [17]. However, the concept of data ethics remains poorly defined, leading to overlapping responsibilities and ambiguous accountabilities. This lack of clarity can hinder the effective detection of ethical violations in data use, as data ethics often merges into broader ethical principles that are insufficient at the granular level of data abstraction [21]. Consequently, there is a need for specific job roles focused on how data ethics can inform ethical decisions regarding data and the machine learning algorithms that process it. These roles would involve establishing relevant policies and practices and advocating for the consumer—here, the patient. Thus, the recommendation to establish a Chief Data Ethics Officer role emerges [21,41]. The mandate of this officer is ethical rather than legal [41], focusing on leading a support team responsible for drafting a code of data ethics, forming and managing a data ethics committee, and overseeing data-oriented ethical issues [21].

With a data-centric organizational culture, data governance, and a Chief Data Ethics Officer, nurse leaders and researchers can explore and implement innovative, nursing-centric data roles. These roles are crucial for upholding the principles of AI data ethics in nursing practice and creating standardized data accountability tools, policies, and processes that effectively evaluate and measure AI data ethics in nurse-specific AI technologies. Job roles such as Chief Nurse Data Ethics Officer and Nurse Data Steward should be explored further.

Nurse educators should implement courses that prepare nurses for data-oriented roles in AI. Nursing degree programs at both undergraduate and graduate levels should be adapted to include relevant courses on AI, data ethics, foundational data science, and data literacy. Nurse scientists are encouraged to explore novel approaches, frameworks, and instruments that enable the integration of ethical accountability mechanisms and effectively assess the principles of AI data ethics reinforced by scientific evidence.

### Recommendation 3: Optimal Data Quality Is Conditioned on Domain Expertise Participation

Domain expertise is essential for generating high-quality data [32], and human involvement is critical to successfully executing data-centric AI tasks [29]. Nurses can use their knowledge of data-centric AI and data ethics to actively engage in all phases of AI development and positively leverage their expertise. Specifically, nurse informaticists have opportunities to participate in activities such as data annotation, labeling, and the verification and validation of data elements used in AI data sets. Furthermore, throughout the AI development lifecycle, nurses can play a pivotal role in identifying and addressing opaque data decisions that may affect frontline clinicians’ trust and usage of AI technologies, thereby enhancing transparency in decision-making processes. The active involvement of nurses in these data-centric AI activities, combined with their commitment to patient advocacy, supports the ethical principles of data privacy and promotes accountability and ownership of data collection, processing, and use.

### Recommendation 4: High-Quality, Ethical Data Curtails Health Care Political Consequences

Nurse leaders, educators, and researchers must recognize that implementing responsible AI technologies and initiatives in health care is complex and challenging, despite significant interest in AI ethics [5]. This statement is not intended to deter nurses from exploring methods to develop ethical AI technologies that use high-quality, representative clinical data. Instead, it aims to raise awareness and encourage nurses to persevere through challenges, including maintaining patience and resilience during the change management process. AI technologies are often politically influenced, reflecting their designer’s values, beliefs, and norms, as well as the data and data sets used to train them [5,42]. Political consequences may arise from data operations such as selection, labeling, preprocessing, and transformation [42]. Nurses, working at various touchpoints across diverse settings in the health care ecosystem, bring unique perspectives to the AI discussion, which can help promote and advocate for the principles of AI data ethics.

### Conclusions

Working with data is time-consuming and challenging and often perceived as less exciting than developing machine learning models or AI technologies. Despite this, the foundational role of data in AI systems cannot be overstated; high-quality data are crucial for the performance and value of AI technologies in health care. The principles of AI data ethics aim to promote responsible AI and the creation of ethical AI technologies [8]. The methods used to collect, store, use, and share data have profound implications for individuals, organizations, and society [8].

## Abbreviations

AI: artificial intelligence
EHR: electronic health record
